# 

**Published:** 1905-12

**Authors:** 


The Passing of Quarantine.

VOL. XXI. DECEMBER, 1905. No. 6.

### TEXAS

### Medical Journal.

SUBSCRIPTION, $1.00 A YEAR, IN ADVANCE.

SINGLE COPIES, 10 CENTS.

PUBLISHED AT AUSTIN, TEXAS, BY

F. E. DANIEL, M. D.,

PROPRIETOR.

PRINTED BY THE VON BOECKMANN-JONES CO.

Entered at the Postoffice at Austin, Texas, as Second-class Mail Matter.

THE "RED BACK"

TWENTY-FIRST YEAR.

